# Forcing closure suggests stronger aerosol forcing and higher climate sensitivity than the consensus

**DOI:** 10.1126/sciadv.aeh6401

**Published:** 2026-10-16

**Authors:** Tianle Yuan, Hua Song, Senne Van Loon, Ryan Kramer, Lazaros Oreopoulos, Piers M. Forster

**Affiliations:** ^1^ Earth Sciences Division, NASA Goddard Space Flight Center, Greenbelt, MD 20771, USA.; ^2^ GESTAR-II, University of Maryland, Baltimore County, Baltimore, MD 21228, USA.; ^3^ Science & Technology Corporation, Columbia, MD 21046, USA.; ^4^ Department of Atmospheric Science, Colorado State University, Fort Collins, CO 80523, USA.; ^5^ Geophysical Fluid Dynamics Laboratory, NOAA, US Department of Commerce, Princeton, NJ 08540, USA.; ^6^ Priestley Centre for Climate Futures, University of Leeds, Woodhouse, LS2 9JT Leeds, UK.

## Abstract

Forcing together with radiative responses and natural variability has sharply increased Earth’s energy imbalance (EEI), a key driver of climate change, between 2003 and 2023. Declining anthropogenic aerosol emissions and increasing greenhouse gas concentrations are the leading contributors to the forcing. However, observation-based and model-based forcing estimates disagree on the magnitude of aerosol forcing, particularly for effective radiative forcing from aerosol-cloud interactions (ERF_ACI_). Here, we show that two independent observation-based estimates of forcing trends achieve unexpectedly good closure between 2003 and 2023. The corresponding model-based trend is within the uncertainty range of observations but substantially smaller, with most of the difference due to models’ underestimation of cloud fraction adjustment to aerosols. Our results suggest a prominent role of aerosols in the recent EEI increase and stronger total historical ERF_ACI_ and higher equilibrium climate sensitivity than the current consensus, with a likely range between 2.8° and 4.8°C.

## INTRODUCTION

Earth’s energy imbalance (EEI) (*N*) is the difference between absorbed solar radiation (ASR) and emitted outgoing longwave (LW) radiation (*1*, *2*). It can also be expressed as the sum of the effective radiative forcing (ERF; *F*) and radiative response to surface warming (*R*)

N=F+R (1)

A positive EEI indicates that Earth is gaining heat, which leads to higher global mean temperature, sea level rise, and melting of ice and snow. Greenhouse gases (GHGs) increase EEI by reducing outgoing LW radiation, which can further trigger changes in planet properties such as global mean surface temperature, cloud radiative effect (CRE), water vapor, and surface albedo, further affecting EEI (*3*). Aerosol particles can affect EEI by producing an ERF (ERF_aerosol_) (*4*) through directly interacting with solar radiation and indirectly modifying cloud properties. Internal variability and changes in natural aerosol and solar irradiance can also affect EEI, but their long-term contribution is much smaller (*1*–*3*, *5*).

Satellite observations from NASA’s Clouds and Earth’s Radiant Energy System (CERES) instrument (*6*) have shown a positive global EEI trend of 0.51 W m^−2^ decade^−1^ for the period of 2003 to 2023 (*3*, *7*, *8*). This positive EEI trend is corroborated by ocean heat content measurements (*9*, *10*). These independent measurements serve as robust constraints on the EEI changes during the CERES period, particularly since 2003, when observations from both the Terra and Aqua satellites became available. The contributions from factors such as clouds, water vapor, surface albedo, GHGs, and global mean surface temperature to the increase in EEI can be estimated (*5*, *7*, *11*–*13*). Natural variability alone has been ruled out as a driver of the observed EEI trend (*5*).

Partitioning the observed EEI into *F* and *R* remains challenging despite its important implications for understanding decadal climate change and climate sensitivity and feedback (*4*, *14*–*16*). The model-calibrated estimates of the forcing trend suggest a 0.51 W m^−2^ decade^−1^ trend for *F* (5 to 95% confidence range of 0.36 to 0.68 W m^−2^ decade^−1^), which would suggest a weak total radiative response trend of −0.01 W m^−2^ decade^−1^ (*16*, *17*) between 2001 and 2023. An observation-constrained estimate using machine learning predicts a stronger negative trend in *R* at −0.21±0.10 W m^−2^ decade^−1^ and a stronger trend in *F* at +0.71±0.21 W m^−2^ decade^−1^ (*16*) between 2001 and 2024. The observed trend in outgoing LW radiation is explained by changes in GHG forcing and radiative response estimates from a radiative kernel method (*11*). The role of internal variability is unable to explain the observed change (*5*). The forcing that drives the increase in the ASR is more uncertain.

Reductions in aerosol emissions in recent decades (Fig. 1) have been shown to substantially increase EEI in several global models (*18*, *19*), whereas multimodel means of a larger collection of models suggest a more muted role of aerosol forcing (*5*, *20*). Analyses of observations and reanalysis data also show a large range of ERF_aerosol_ in driving the recent EEI increase with some showing a substantial contribution from ERF_aerosol_ (*18*, *19*, *21*–*24*) and others indicating a limited contribution (*25*, *26*). The divergent results on the impact of aerosols between different studies reflect the overall high uncertainty of ERF_aerosol_ since the preindustrial era (*4*, *27*). The large uncertainty is partially due to differences in the representation of aerosols and cloud microphysics in global models and in how observational data are analyzed (*22*, *27*–*29*). The most important source of uncertainty is ERF from aerosol-cloud interactions (ERF_ACI_) because global models and observation-based radiative kernel calculations agree on a trend of around +0.06 W m^−2^ decade^−1^ in the forcing from aerosol-radiation interactions (ARIs) (*11*, *18*), which is too weak to explain the observed increase in ASR by CERES. Changes in clouds, both due to feedback and aerosol-cloud interaction (ACI), must thus have a central role in explaining the ASR trend (*7*, *13*, *15*). An improved quantification of either contribution would help to constrain the other, reducing the uncertainty in both.

**Fig. 1.**
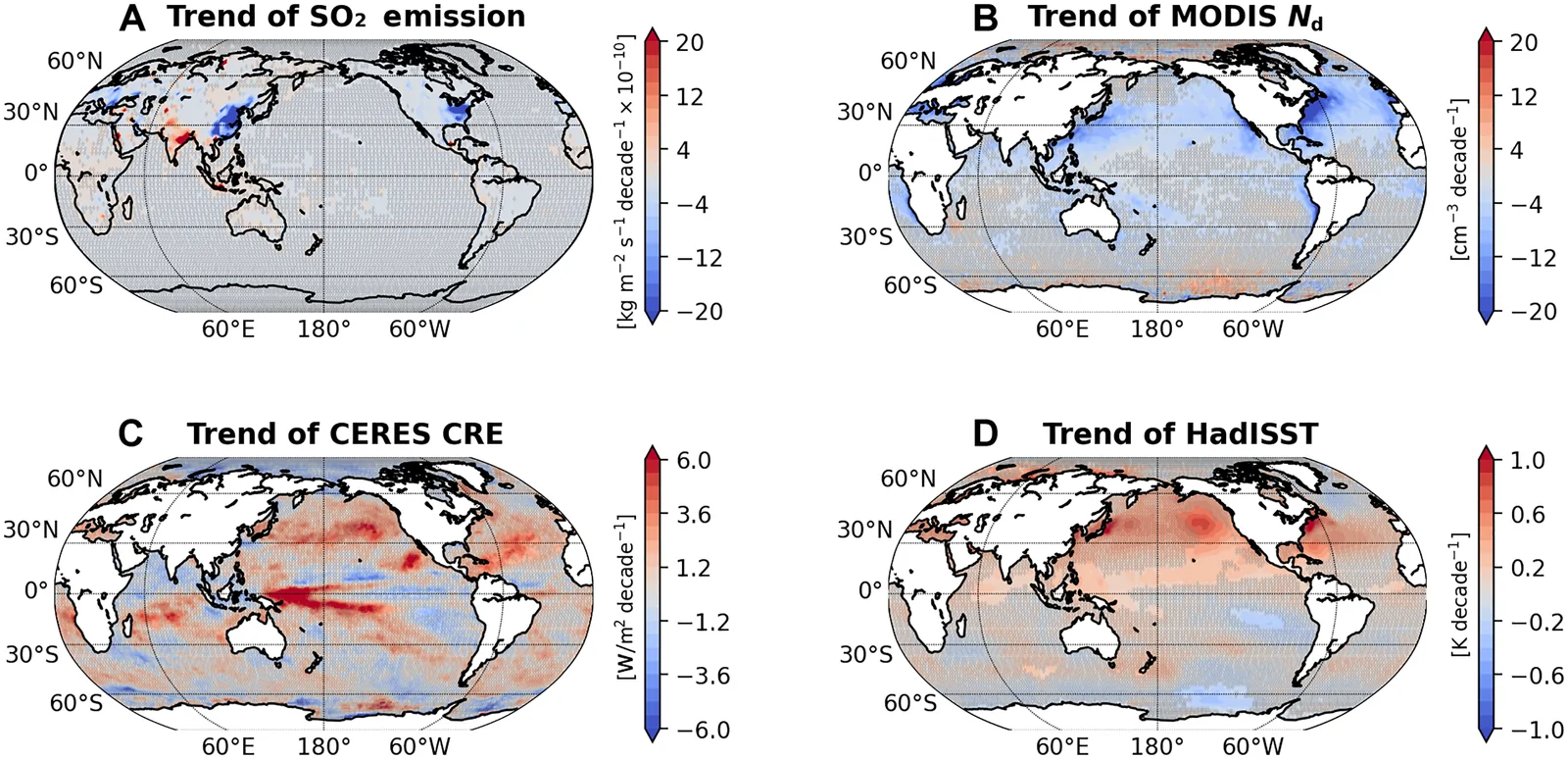
Trend maps of SO_2_ emissions, *N*_d_, CRE, and SST. Trend maps of (**A**) $SO_{2}$ emissions from (*30*), (**B**) MODIS $N_{d}$ for the same period, and (**C**) CERES SW CRE trend between 2003 and 2023. Positive values indicate that less solar energy is reflected back to space by clouds. (**D**) SST from HadISST. Stippled regions are for *P* > 0.05 (*58*).

Here, we perform a closure study by comparing the trends in *F* from two independent estimates between 2003 and 2023 to assess the role of ERF_ACI_ during both this recent and historical periods. In one method, we quantify ERF_ACI_ by applying cloud adjustment functions from ship-track analyses on satellite data (*21*, *29*) (see Materials and Methods for more details and fig. S1). We then combine this ERF_ACI_ with other forcing components (*11*, *17*) to obtain the trend in total *F*, which we call “bottom-up” estimate, $F_{\text{BU}}$. We then compare $F_{\text{BU}}$ with the forcing trend estimated by subtracting *R* from the observed EEI trend, which we call “top-down” estimate, $F_{\text{TD}}$ (*16*). The top-down approach uses EEI and surface temperature observations and only relies on climate models to predict the relationship between surface temperature and *R*, which makes it methodologically orthogonal to the bottom-up estimate (see Materials and Methods for more details). Together with the best model estimates, we diagnose the source of disagreements and discuss the implications.

## RESULTS

### Observed trends in aerosols, clouds, and SST

Figure 1 shows the geographical distribution of trends of $SO_{2}$ emissions [between 2003 and 2022 (*30*)], cloud droplet number concentrations ($N_{d}$), shortwave (SW) CRE from the CERES EBAF data, and sea surface temperature (SST) between 2003 and 2023. Statistically insignificant trends at the 95% confidence level are stippled. The $SO_{2}$ emission trend map (Fig. 1A) shows strong reductions over East Asia, North America, and Western Europe as expected from recent aerosol reductions in those regions (fig. S2), with emissions down by more than 80% over North America and Western Europe (fig. S3). The strong negative trend in East Asia following an initial positive trend resulted in an overall negative trend. The effects of reduced aerosol loading can be directly observed in the trends of MODIS aerosol optical depth (AOD; fig. S4). Trends in MODIS *N*_d_ are consistent with the emission changes (Fig. 1B). Strong decreases in anthropogenic $SO_{2}$ emissions in regions like East Asia, North America, and Western Europe (*19*, *24*) have reduced the number of cloud condensation nuclei and thus *N*_d_ over oceans downwind of these regions. Negative $N_{d}$ trends affect cloud properties and are expected to increase SW CRE due to ACIs.

SW CRE has increased or trended less negative, i.e., reflecting less sunlight back to space, over many areas (Fig. 1C), consistent with previous studies (*7*, *13*). This increase in CRE is an important contributor to the increasing EEI trend (*7*, *13*). The positive trend in CRE is especially strong in the North Pacific and the North Atlantic where low clouds are widespread. Because of reduced aerosols and $N_{d}$, both the Twomey effect and the cloud fraction (CF) adjustment would increase CRE through decreased cloud albedo and fraction (*31*, *32*). The observed increase in CRE can also be due to cloud feedback (*13*, *33*). We show trends of SST in Fig. 1D to illustrate that they can also be driving the trends in CRE. Over the same regions in the North Pacific and North Atlantic where a strong increase in CRE is observed, SST trends are positive, which is consistent with the effect of SST on CRE (*13*, *33*). It is worth noting, however, that SST trends over these areas are not always statistically significant at the 95% confidence level (Fig. 1D).

Next, we examine the closure between the bottom-up and the top-down estimates and quantify the contributions of ERF_ACI_ along with those of other forcing agents resolved in the bottom-up method.

### Closure of forcing trends and comparison with models

Figure 2A shows time series of total forcing anomalies between 2003 and 2023 from four different approaches. The Forster *et al.* (*17*) (F25) forcing trend of +0.58±0.05 is from ref. (*17*) and extends the AR6 forcing estimates of (*4*) with updated emissions and model calibrated regressions. The Van Loon *et al.* (*16*) forcing (top-down estimate) shows a stronger trend of +0.73±0.21 W m^−2^ decade^−1^ during this period. Our bottom-up estimate is the average of five values from five different forms of cloud adjustment functions in SW ERF_ACI_ calculations (*29*) (see Materials and Methods for more details). The uncertainty associated with using different cloud adjustment functions is shown as dark gray shades. The bottom-up forcing trend is +0.7±0.15 W m^−2^ decade^−1^, very close to the top-down estimate and stronger than the F25 trend. It is important to note that the confidence intervals of all three estimates still partially overlap. Furthermore, it is worth noting that the bottom-up estimate is not completely independent of the F25 estimate because they differ mainly in ERF_ACI_ estimates.

**Fig. 2.**
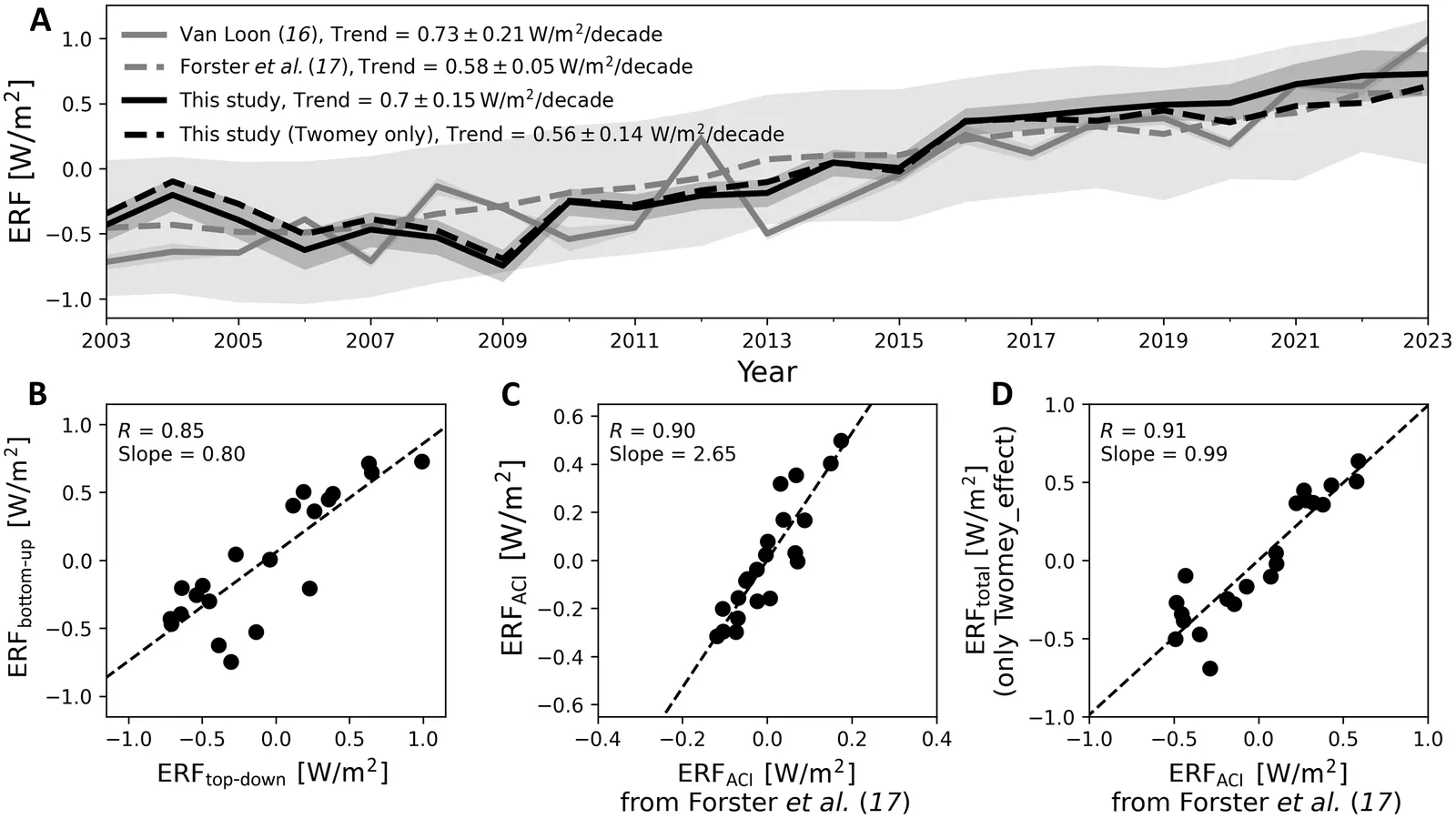
Time series of forcing anomalies from four methods and their relationship. (**A**) Trends of four approaches including the top-down, bottom-up, multimodel mean, and bottom-up but only accounting for the Twomey effect in ERF_ACI_; the gray shading show the confidence interval of F25. (**B**) Correlation between the top-down and bottom-up forcing estimates. (**C**) Correlation between annual bottom-up and multimodel mean estimates. (**D**) Same as (C) but with only the Twomey effect in the bottom-up estimate.

The fact that the forcing trend estimates of the bottom-up and top-down methods are statistically indistinguishable and differ by only 0.03 W m^−2^ decade^−1^ is nontrivial. First, the two methods are largely independent in their primary constraints, datasets, and methods (see Materials and Methods and fig. S1). The top-down method estimates the forcing by removing *R* based on observed surface temperature maps in Eq. 1 from the CERES observed EEI. The bottom-up method relies on cloud adjustment functions and MODIS observations of cloud properties for calculating ERF_ACI_. Second, SW ERF_ACI_ is uncertain and likely the dominant factor that contributes to the difference in forcing trends between different methods because both GHG forcing and ERF from ARIs can be well accounted for (*11*, *16*–*19*, *21*, *25*, *27*). The top-down method does not have explicit information of processes regarding ERF_ACI_ but rather estimates the total *F* from EEI by removing radiative responses. The two estimates are also highly correlated with a correlation coefficient of 0.85 (Fig. 2B). However, most of the correlation stems from the common trend. After detrending the data, the correlation coefficient becomes statistically insignificant, suggesting that the two do not agree on the detrended interannual variations yet.

Given that the trend of ERFGHG is around +0.38±0.14 W m^−2^ decade^−1^ (from F25), a substantial part of the trend in total forcing comes from ERF_ACI_ (*18*, *19*, *21*) if we assume that the top-down and bottom-up estimates are closer to the true values. Our bottom-up calculations suggest that SW ERF_ACI_ has a trend of +0.37 W m^−2^ decade^−1^ between 2003 and 2023 using five alternative cloud adjustment functions, with a spread of 0.07 W m^−2^ decade^−1^. This spread only characterizes the sensitivity of the trend to the adjustment formulations considered here and should not be interpreted as a comprehensive uncertainty estimate for ERF_ACI_ (see Materials and Methods). For comparison, the SW ERF_aerosol_ trend is +0.21±0.07 W m^−2^ decade^−1^ between 2001 and 2019 based on four model simulations (*18*), whereas our estimate is +0.31±0.07 W m^−2^ decade^−1^ between 2003 and 2019, which is stronger than the multimodel mean value by +0.1 W m^−2^ decade^−1^ although still within the range of model-simulated trends. The multimodel mean trends in SW, after taking aerosol forcing into account, still underestimate the CERES SW trend by around 0.2 W m^−2^ decade^−1^ between 2001 and 2019 (*18*). Simply replacing the model SW ERF_ACI_ trend with our bottom-up estimate of the SW ERF_ACI_ trend would help to close this gap by 0.1 W m^−2^ decade^−1^ or 50%. The top-down and bottom-up estimates being close to the true value would suggest that current multimodel means may be underestimating SW ERF_aerosol_ during this period, which is consistent with recent studies (*19*, *34*).

We find a strong correlation between ERF_ACI_ from F25 and our bottom-up estimates (Fig. 2C), despite the two trends differing by almost 0.24 W m^−2^ decade^−1^. The estimate of ERF_ACI_ in F25 is calibrated by results from AR6 models and scaled through simulated *N*_d_ that are ultimately related to aerosol emissions (*17*). The close correlation between the two ERF_ACI_ time series suggests that the models perform well in capturing the temporal variations of $N_{d}$ driven by emission changes when compared to MODIS retrievals. Similarly, radiative kernel results show an ERFARI trend that is in close agreement with the model simulated trend at +0.06 W m^−2^ decade^−1^ (*11*, *18*), again suggesting that models can capture the observed decrease in aerosol loading due to decreased emissions. These results suggest models simulate well the aerosol changes but underestimate ERF_ACI_ trends, i.e., the impact of aerosols on clouds, compared to both the top-down and bottom-up estimates.

### Breakdown of ERF_ACI_

We break down the total ERF_ACI_ from our bottom-up method into three different contributions: the Twomey effect, the liquid water path (LWP) adjustment, and the CF adjustment (Fig. 3A). Our calculation suggests that the CF adjustment contributes +0.2±0.03 W m^−2^ decade^−1^ to the trend, whereas the LWP adjustments adds only −0.03±0.01 W m^−2^ decade^−1^ during 2003 to 2023. The magnitude of the CF adjustment effect is very close to that of the Twomey effect. We show forcing trends from F25, bottom-up total, and bottom-up with only the Twomey effect for ERF_ACI_ in Fig. 3B. If only the Twomey effect is included in calculations of bottom-up forcing, the forcing trend becomes +0.56±0.14 W m^−2^ decade^−1^, which is very close to the F25 estimate of +0.58 W m^−2^ decade^−1^ (Fig. 4). The correlation coefficient between these two forcing estimates is 0.9 with a slope of 0.99 (Fig. 2D). Our results thus suggest that the extra warming effect in our bottom-up estimate compared to the F25 may be mainly due to the CF adjustment because the LWP adjustment has a negligible contribution to the global mean [see Fig. 3 and (*21*, *29*, *35*)] and the Twomey only estimates agree well with F25.

**Fig. 3.**
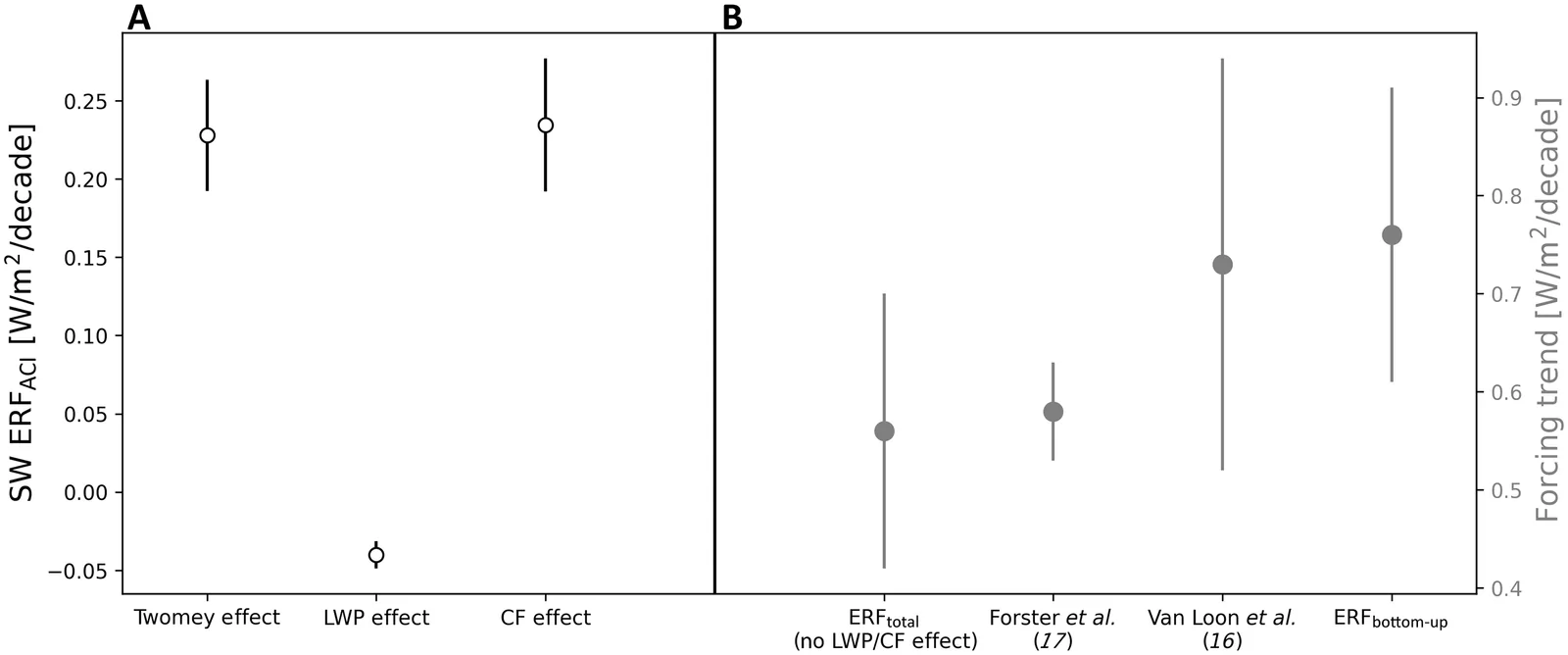
Breakdown of aerosol forcing into three components. (**A**) Breakdown of SW ERF_ACI_ trends into three components: the Twomey effect, the LWP adjustment, and CF adjustment. (**B**) Total forcing trends for four different methods (same as the values in Fig. 2) (*16*, *17*). The CF adjustment contributes roughly the same warming effect as the Twomey effect, whereas the LWP adjustment produces a small cooling effect. When only the Twomey effect is considered, the total forcing trend from the bottom-up method matches well the F25 trend.

**Fig. 4.**
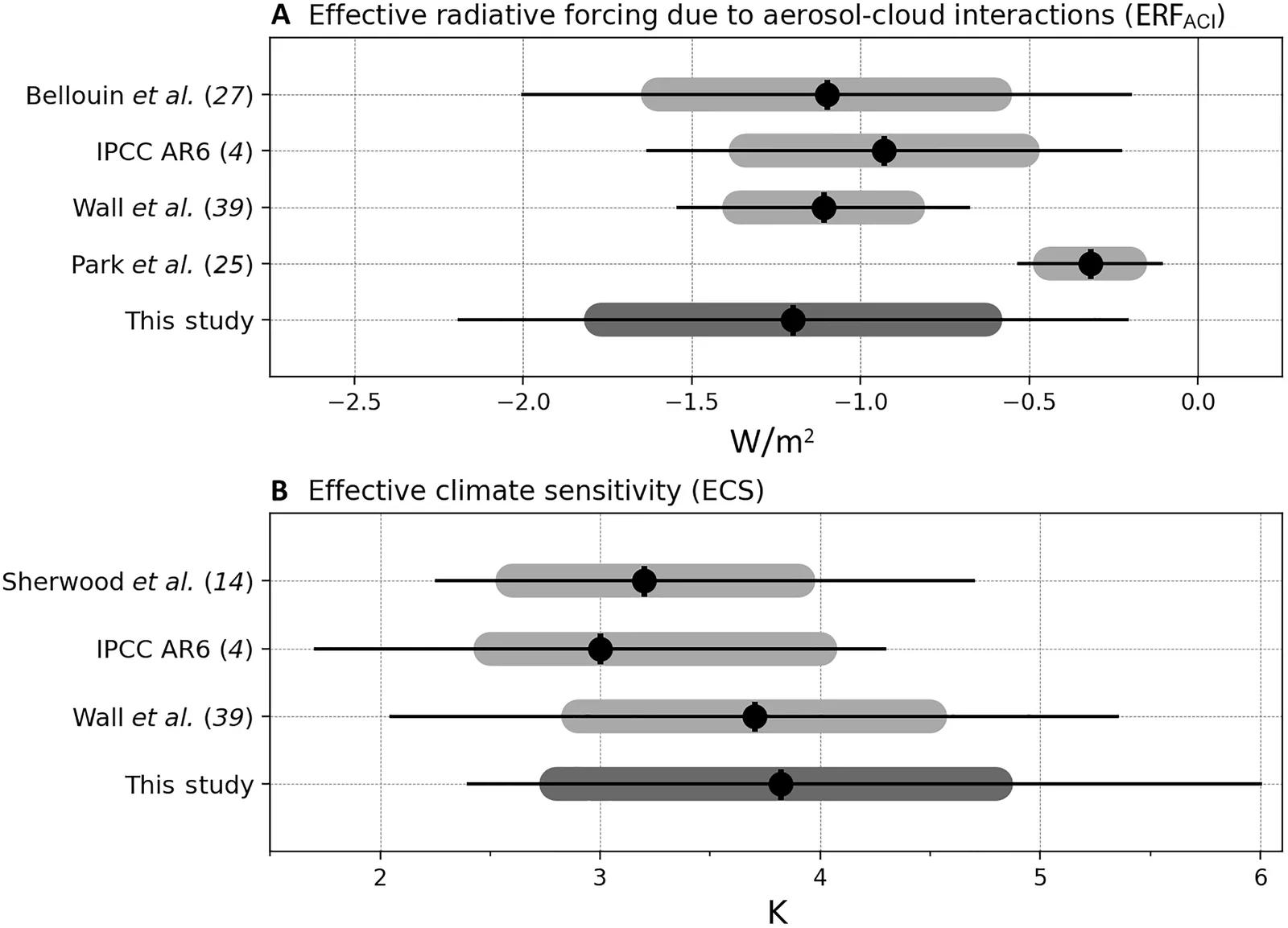
Estimates of ERF_ACI_ and implied ECS from this study compared to previous studies. (**A**) Global mean ERF_ACI_ and (**B**) ECS from our study and previous studies (*4*, *14*, *25*, *27*, *39*). Thin lines and rounded blobs represent the 90% (5 to 95%) and 66% (17 to 83%) confidence intervals with the central estimate marked as black dots. The studies were sorted by their ERF_ACI_ and ECS magnitudes.

## DISCUSSION

The trends from the independent bottom-up and top-down methods being close to the true value would have important implications. First, it would mean that the overall downward trend of aerosol emissions has contributed around +0.37 W m^−2^ decade^−1^ to the increase in EEI during the same period. The climate models in F25 capture both the ERFARI trend and the temporal variations of the ERF_ACI_ as shown in the Results section, suggesting skill in converting emission changes into aerosol loading variations. However, they underestimate ERF_ACI_ due to underestimating CF adjustment as discussed later. The strong ERF_ACI_ in this study is driven mostly by emission reductions in recent decades and has important implications for solar radiation management studies and near-term warming (*21*). Assuming a linear scaling between $\Delta N_{d}$ and ERF_ACI_ (*36*, *37*) and that low clouds are not saturated with aerosols at the beginning of our period, our results suggest a higher sensitivity of ERF_ACI_ to $\Delta N_{d}$ and thus stronger overall ERF_ACI_ between preindustrial and present day than the multimodel mean. It is important to note that our central estimate of ERF_ACI_ is still within the range of model simulations (*18*, *27*) but close to the high end of the model ensemble (see also Fig. 4).

Second, a stronger magnitude of ERF_ACI_ suggests a smaller magnitude of total ERF because the positive GHG forcing is compensated by the stronger negative ERF_ACI_. The observed temperature increase because the preindustrial era is thus a result of a smaller net positive forcing, implying a higher historical effective climate sensitivity. This agrees with conclusions of an independent study that interrogated model simulations with observed EEI with a different method (*15*). We apply the same bottom-up method to calculate $\mathrm{\Delta ER}F_{\text{ACI}}$ between 1850 and 2010 using data from (*27*) to obtain $\Delta N_{d}$ for this period (*29*). Our estimated $\mathrm{\Delta ER}F_{\text{ACI}}$ is $-1.2\pm 0.6\;W\;m^{-2}$ (Fig. 4A) between 2005 to 2015 and 1850 to 1860, stronger than the mean values from previous estimates (*4*, *22*, *27*, *28*, *38*). Our central estimate of $\mathrm{\Delta ER}F_{\text{ACI}}$ is closer to those from (*27*) and (*39*) and near the 83rd percentile of estimates based on a hemispheric contrast constraint (*28*).

We examine the implications of such ERF_ACI_ values for equilibrium climate sensitivity (ECS) using the Bayesian framework that considers evidence from paleoclimate, historical, and process understanding studies to produce a posterior ECS distribution (*14*). Following (*14*, *39*), we replace expert-assessed ERF_ACI_ values with our estimates and keep everything else unchanged. The resulting posterior ECSs have a mean of 3.8°C with a likely range (66% confidence interval) of 2.8° to 4.8°C (Fig. 4B). Our central estimate of ECS is higher than both the IPCC AR6 (2.5° to 4.0°C) and WCRP (2.6° to 3.9°C) values by about 0.5°C, and our ECSs have a wider range (*14*, *40*). The central estimate agrees well with that from ref. (*39*) (2.9° to 4.5°C) (Fig. 4B). Nevertheless, there is significant overlap between our estimates of ERF_ACI_ and ECS and previous studies (Fig. 4). Our values are the closest to those from (*39*) that are also based on satellite data but on a different method. Our ECS range indicates more than 22% chance of ECS being higher than 4.5°C [versus 17% and 7% for refs. (*39*) and (*14*), respectively].

Our breakdown of ERF_ACI_ suggests an important role of low CF adjustment to aerosols between 2003 and 2023 because its contribution almost rivals that of the Twomey effect and many global models may underestimate this effect. This agrees with recent studies that show many current models underestimate this CF adjustment effect (*19*, *29*, *34*, *41*). For example, Wang *et al.* (*34*) show that current global models do not capture the increase in CF due to the addition of aerosols from effusive low lying volcanoes as seen in observations. Modeling experiments with an improved parameterization scheme show that CF adjustments contribute even more forcing to the Twomey effect in the North Atlantic and Northeast Pacific regions during similar periods (*19*). Our results also suggest that, although models are capable of simulating the temporal aerosol variations induced by emission changes, they may not produce enough forcing from the CF adjustment. Our results, as well as previous observation-based studies (*29*, *39*), suggest that CF adjustment plays a major role in ERF_ACI_, whereas the LWP adjustment contributes minimally (*29*, *35*). Models show a wide range of values for the ratios among the three components, and they need to be better constrained (*19*, *27*, *42*, *43*). The strength of the CF adjustment also has important implications for the effectiveness of marine cloud brightening.

The agreement between the top-down and bottom-up methods suggest that *R* derived from the top-down method is not dominated by noise large enough to obscure the forcing signals at annual time scales (*16*). The temporally stable *R* estimate is attractive, although the method does not constrain the absolute value of *R*. Further refinement and calibration of the *R* estimates would help reduce the uncertainty range of ERF_ACI_. The agreement between the two methods further suggests that the radiative response term has an overall stabilizing effect during 2003 to 2023 at around −0.2 W m^−2^ decade^−1^ (*16*). The top-down estimate of *F* is also critically dependent on the availability of long-term and stable spaceborne observations of EEI (*3*, *44*). Such observations anchor the partition between forcing and radiative responses for both bottom-up and top-down methods and provide a potential means to reduce uncertainty in ERF_ACI_.

Although the good closure between the top-down and bottom-up calculations is encouraging and adds confidence to the results from both methods, we must emphasize that it does not assure the accuracy of the ERF_ACI_ values, which are substantially uncertain even in our bottom-up estimates. For example, the SD of the ERF_ACI_ trends is 0.15 W m^−2^ decade^−1^ due to the choice of cloud adjustment functions (Fig. 2A) (*29*). Most of this uncertainty comes from the CF adjustment contribution (Figs. 3 and 4), which needs to be more rigorously constrained (*19*, *29*, *34*). Our study adds another data point in estimating ERF_ACI_, and further investigations are required to reconcile the different results on the magnitude of ERF_ACI_ from preindustrial to present (*20*, *25*, *27*, *28*, *36*–*39*).

Two independent methods we call top-down and bottom-up, which are based on satellite observations, agree well in estimates of ERF trend between 2003 and 2023. Although this closure does not prove that our estimates are the true values, it increases confidence in both. Our results suggest the recent decrease in aerosol emissions has contributed roughly equally to the forcing as the GHGs. Using the same bottom-up method, we obtain stronger ERF from aerosols than previous estimates, implying a climate sensitivity around 3.8°C, which exceeds current expert-assessed values. The modeled forcing trends are lower than by around 0.14 W m^−2^ decade^−1^ but within the uncertainties of the observational estimates. Disagreement in the contribution of the ERF from ACIs is the most likely reason for the difference between our estimates and those from models. More specifically, although models seem to have skill in simulating forcing from ARIs and the Twomey effect, they appear to have too weak CF adjustments. It is critical that future work reconciles the divergent estimates of aerosol forcing over the same era from different studies given its broad implications such climate sensitivity, climate feedback, and solar radiation management.

## MATERIALS AND METHODS

### Data

We use Level-3 Aqua MODIS products (*45*) in this study, which provide cloud optical depth and cloud droplet effective radius resolved at 1° after aggregation of pixel level values. $N_{d}$ and LWP are derived from these two retrievals. For $N_{d}$, we adopt the approach of (*46*). In the main text, we report results using equation 11 from (*46*) to derive $N_{d}$ (*21*, *47*, *48*). Radiative fluxes come from the CERES Energy Balanced and Filled (EBAF) Edition 4.2 products. They include SW reflected fluxes, outgoing LW radiation fluxes, both SW and LW CREs, and cloud albedo. EEI is derived from EBAF variables (*6*). Forcing estimates from F25 (*17*) used here to compare bottom-up and top-down estimates are publicly available.

The top-down method uses a combination of large initial-condition ensemble climate model simulations (CanESM5, IPSL-CM6A-LR, MIROC6, and MPI-ESM1.1) and surface temperature reconstructions (ERA5, COBE2, NOAAGlobalTemp 6.0.0, and HadISST-1.1) to estimate *R*. See below and (*16*) for more details.

### Methods

#### Forcing closure framework

We interpret variations in Earth’s top-of-atmosphere (TOA) radiation in terms of a standard global energy balance framework. The planetary energy imbalance N is written as Eq. 1. F is the ERF from external drivers, and R is the net radiative response of the climate system to surface temperature changes.

In this study, we seek a closure between (i) a “top-down” estimate of total ERF, $F_{\text{top}}$, time series inferred from observed energy imbalance and estimated radiative response and (ii) a “bottom-up” reconstruction, $F_{\text{BU}}$, obtained by summing individual forcing components derived from radiative kernels, multimodel estimates, and satellite-based ERF_ACI_ estimates. The closure is illustrated in the flowchart in fig. S3.

Both methods use CERES data but in a different way. The top-down method uses the global mean EEI record from CERES and SST data to obtain *R*. The bottom-up method uses estimates of gridded surface albedo and cloud albedo as well as incoming downwelling solar flux data together with MODIS data to calculate forcing first at the grid cell level and then as a global mean average. Therefore, there is no structural reason for these two methods to agree on the forcing because both their data and methodology are independent.

#### Top-down forcing estimate

The top-down forcing estimate, $F_{\text{top}}$, is obtained by subtracting R from CERES-derived N. The method is described in detail in ref. (*16*) and summarized briefly here. First, we train a convolutional neural network (CNN) to predict global mean R based on spatial SST maps on yearly timescales. The CNN is trained on large ensemble climate model simulations with the ensemble mean removed. By removing the ensemble mean, the training data only contains internal variability within climate models. Thus, the CNN never sees a forced response during training. Then, the CNN is applied to observation-based SST reconstructions to estimate R in the historical record. Last, we subtract the CNN-predicted R from CERES-derived N to produce an observation-based estimate of ERF $F_{\text{top}}=N-R$. The uncertainty of this estimate is likely underestimated because it neglects uncertainties related to the machine learning method or training data.

The CNN is nonlinear and takes into account the pattern effect on radiative feedbacks because it takes SST maps as input. That is, the CNN has learned that R varies with the spatiotemporal SST pattern, not only the global mean temperature, and the CNN is able to predict the historically varying feedback (*16*). Although the CNN is trained on internal variability only, it is able to predict the response to SST changes in historical model simulations that include a forced component (i.e., a warming climate). When applying the CNN to historically warming SST patterns predicted by climate models, it correctly reproduces the forced radiative response R, both in climate models it was trained on and in climate models it has never seen during training, validating the approach [see figure 3 in (*16*)]. Moreover, gradients of the CNN indicate that the CNN has learned a physically meaningful relationship between SST and R [figure 2 in (*16*)]. Because of this, we expect the CNN to extrapolate to observed SST, although we do not know the true, observed, R.

Because the CNN-predicted R contains only temperature-mediated feedbacks, $F_{\text{top}}$ includes all external forcing agents (both anthropogenic and natural) and rapid adjustments in the stratosphere and troposphere (*4*). We can write

$$\begin{array}{c} F_{\text{top}}\approx \text{ER}F_{\text{GHG}}+\text{ER}F_{\text{ACI}}+\text{ER}F_{\text{ARI}}+\text{ER}F_{O_{3}}\\ +\text{ER}F_{\text{LU}}+\text{ER}F_{\text{other anthro}}+\text{ER}F_{\text{natural}} \end{array}$$ (2)

where the terms denote ERF from well-mixed GHGs, aerosols (ARI plus ACI), ozone, land-use/surface-albedo changes, other anthropogenic contributions (e.g., aviation and stratospheric water vapor), and natural forcings (solar and volcanic), respectively.

#### Bottom-up forcing estimate

Our bottom-up construction aims to estimate each component in Eq. 2. Instantaneous radiative forcing (IRF) of GHG comes from ref. (*11*). So does the $\text{IR}F_{\text{ARI}}$. $\text{IR}F_{\text{ACI}}$ is based on satellite observations and cloud adjustment functions as detailed later (*21*, *29*). To close the budget with the top-down ERF, we also account for rapid adjustments of GHG forcing, non-cloud rapid adjustments, ozone forcing, land-use forcing, other anthropogenic contributions, or natural forcings. ERF from ozone, land-use, other anthropogenic agents, and natural forcings come from ref. (*17*). The total $\text{ER}F_{\text{aerosols}}$ contains IRF from ARI and ACI and non-cloud rapid adjustments. We account for the adjustment by including a +30% for the $\text{IR}F_{\text{aerosols}}$ to span the range that is potentially induced by the adjustment (*4*). This 30% range is larger than what is reported in the literature but chosen to be conservative so that we can safely account for all potential range. Following the results from (*22*), we assume a LW forcing component of forcing from the ACI that is 14% of the SW part but with a different sign. Considering this LW component reduces the historical total ERF_ACI_. We approximate the transformation from IRF to ERF for GHGs using scaling factors diagnosed from climate models. We write

$$\text{ER}F_{\text{GHG}}\approx \left(1+\alpha_{\text{GHG}}\right)\;{\text{IRF}}_{\text{GHG}}$$ (3)

where $\alpha_{\text{GHG}}$ is a model-derived factor that accounts for stratospheric and other rapid adjustments to GHG forcing. We choose a value of 0.15 for $\alpha_{\text{GHG}}$ (*49*).

We therefore have $F_{\text{BU}}$ as

$$\begin{array}{c} F_{\text{BU}}\approx \left(1+\alpha_{\text{GHG}}\right)\;{\text{IRF}}_{\text{GHG}}+{\text{ERF}}_{\text{aerosols}}+\text{ER}F_{O_{3}}\\ +\text{ER}F_{\text{LU}}+\text{ER}F_{\text{other anthro}}+\text{ER}F_{\text{natural}} \end{array}$$ (4)

Equations 2 and 4 provide a closed bottom-up ERF estimate that can be directly compared to the top-down ERF, $F_{\text{top}}$, enabling a quantitative assessment of consistency between observationally constrained forcing components and the total forcing required to explain the observed energy imbalance. Other terms in *F*_BU_ come from Forster *et al.* (*17*).

#### Cloud adjustment functions

Cloud adjustment functions are derived from analyses of numerous ship-track samples (*29*). We automatically contrast background cloud pixels and ship-track pixels for each ship-track segment. We consider the effect of cloud overlap between high and low clouds when calculating the difference of CF (*29*). Only low cloud pixels are included for calculating the average of cloud properties. For each segment, we calculate changes in LWP, $N_{d}$, CF, and $r_{e}$ between ship-track and background control pixels. The cloud adjustments are computed to get the sensitivity of CF and LWP to $N_{d}$ as the ratio of changes in CF and LWP over changes in $N_{d}$: $\frac{d\text{ln}\;\text{LWP}}{d\text{ln}\;N_{d}}$ and $\frac{d\text{CF}}{dN_{d}}$.

We then bin cloud adjustments based on different background variables such as control cloud $N_{d}$, background CF, estimate inversion strength, etc. (*21*, *29*). In total, we have five different functional forms to represent a diverse range of possibilities because the true adjustment functions are still uncertain. We show that these five function forms produce $\text{ER}F_{\text{ACI}}$’s that span a wide range (*29*). ERF_ACI_’s calculated from the five adjustment functions represent the range of possibilities for ERF_ACI_ in our study, and we calculate the mean and SDs based on these five results. The derived cloud adjustments are robust against random sample shuffling such as selecting samples from different periods or randomly remove 50% of the samples.

#### Calculating ERF_ACI_

We consider the sensitivity of scene albedo [$A=A_{\text{ac}}\;f_{\text{total}}+A_{s}\left(1-f_{\text{total}}\right)$] to $N_{d}$, we have

$$\begin{array}{c} S^{*}=\frac{\mathrm{dA}}{dN_{d}}=\frac{d\left[A_{\text{ac}}\;f_{\text{total}}+A_{s}\left(1-f_{\text{total}}\right)\right]}{dN_{d}}\\ \approx f_{c}\times S+\left(1-f_{\text{high}}\right)\times \frac{df_{c}}{dN_{d}}\times \left(A_{\text{ac}}-A_{s}\right) \end{array}$$ (5)

where *A* is the all-sky scene albedo, i.e., including both cloudy, *A*_ac_, and clear, *A*_s_, parts; $f_{\text{total}}$ and $f_{c}$ are all cloud and low CF obtained from the MYD08_M3 data; *A*_s_ is the surface albedo, derived from the CERES EBAF-TOA data; and $1-f_{\text{high}}$ is used to take into account of effect of overlap on $f_{c}$ adjustment and derived from the MODIS cloud product. S is the sensitivity of cloud albedo to $N_{d}$, including both the Twomey and LWP adjustment effect

$$S=\frac{dA_{c}}{dN_{d}}=\frac{A_{c}\left(1-A_{c}\right)}{3N_{d}}\times \left(1+\frac{5}{2}\frac{d\text{ln}\;\text{LWP}}{d\text{ln}\;N_{d}}\right)$$ (6)

where $A_{c}$ is the cloud albedo. It is approximated from the CERES all-sky albedo ($A_{\text{allsky}}$), clear-sky albedo ($A_{\text{clearsky}}$), and total CF ($f_{\text{total}}$) using the equation

$$A_{c}=\left[A_{\text{allsky}}-\left(1-f_{\text{total}}\right)\times A_{\text{clearsky}}\right]/f_{\text{total}}$$ (7)

We simplified the calculation by approximating cloudy albedo at the top of the atmosphere by that at the cloud top and clear sky albedo by surface albedo because the atmospheric effect on the cloudy and clear sky albedos partially cancels. We assume zero aerosol effects on high CF. $\frac{df_{c}}{dN_{d}}$ is calculated using our ship-track samples, similar to the LWP adjustment. We account for the cloud overlap between high and low clouds. For more details, we refer to our previous studies (*21*, *29*).

We can get changes in TOA flux as the forcing by

$${\text{ERF}}_{\text{ACI}}=-SW_{\text{downwelling}}\times S^{*}\times \Delta N_{d}$$ (8)

which produces forcing from ACI. More detailed discussions about sensitivity and uncertainty of our approach can be found in our previous studies (*21*, *29*).

$N_{d}$ anomalies are produced using MODIS retrievals between 2003 and 2023. For each 1°×1° grid, we remove the climatological mean and the seasonal cycle to derive $\Delta N_{d}$ time series. Using the equations above and the derived $\Delta N_{d}$, we can calculate ERF_ACI_ for each grid point at monthly time scales. Our method of estimating ERF_ACI_ accounts for effects from fast cloud adjustments (LWP and CF). To also account for the non-cloud adjustments, we assess an uncertainty range as discussed in the Bottom-up forcing estimate subsection above.

Our calculated ERF_ACI_’s have been independently compared to other estimates under different scenarios. For example, we estimate the ERF_ACI_ between preindustrial and present day to be $-1.1\pm 0.8\;W\;m^{-2}$, which is close to the consensus number of $-0.84\pm 0.6\;W\;m^{-2}$ in the Sixth Assessment Report (*4*). It is also very similar to an independent estimate ($-1.02\pm 0.43\;W\;m^{-2}$) also based on MODIS data (*22*). We also applied our method to estimate ERF_ACI_ from shipping fuel regulation (*21*); our estimate was very close to values from independent modeling studies at around 0.1 to 0.15 W m^−2^ globally (*48*). ERF_ACI_ from our method is compared to and consistent with independent estimates based on top-down geospatial kriging approach in the southeast Atlantic shipping lane (*21*, *50*). Both methods show around +1 W m^−2^ of warming due to reduced ship emissions in the core of the shipping corridor. These agreements indicate that our method provides reasonable estimates of ERF_ACI_ by applying cloud adjustments from ship-tracks to larger scales. Assumptions made in our method and their impact is discussed in the Assumptions subsection.

#### Historical ERF_ACI_ and ECS estimates

For the historical ERF_ACI_, we use changes of AOD from (*27*) and follow previous studies (*29*, *35*) to convert them into $\Delta N_{d}$. We then use the above method to estimate the change in ERF_ACI_ between 2005 to 2015 and 1850 to 1860. Following (*39*), we replace ERF_ACI_ values from (*14*) with ours and calculate the posterior estimate of ECS with other parameters and data unchanged. The Bayesian framework from (*14*) considers multiple lines of evidence from various sources and runs Monte Carlo simulations to calculate posterior ECS distributions. Our exercise thus only estimates the impact of ERF_ACI_ changes on the resulting ECS and should be considered in that context. Our results add another data point to the range of possibilities and should not be interpreted as a new assessment of the ECS.

#### Assumptions

We note a few assumptions and simplifications inherent to our method. The LW effect of ACIs in low clouds is not considered. This effect is of secondary importance due to the small temperature contrast between low clouds and surface, with a previous study suggesting that it accounts for roughly 10% of the SW ERF_ACI_ (*4*, *22*). The cloud adjustments used here apply best to the oceanic stratocumulus regime because most ship-tracks form in this cloud regime (*51*), but we assume that they also apply to oceanic trade cumulus clouds as well. Sensitivity tests show that if all effects in the trade cumulus regions are assumed to be 0, the forcing would be changed by less than 10% in the global mean (*29*). Comparing results from our method with previous studies suggests that our method estimates smaller CF changes in response to $N_{d}$ than other published studies for the trade cumulus clouds (*52*, *53*), which would make our estimated ERF_ACI_ for these clouds a lower bound. Cloud adjustments to aerosols can evolve significantly with time. The adjustments derived from ship-tracks represent a time-average behavior, which may not be strictly representative because ship-track clouds have different ages (*54*). Other assumptions and uncertainties are discussed in (*29*).

Over land, we only consider the Twomey effect and ignore the cloud adjustments because our adjustment functions are based on ocean data only.

#### Radiative kernel calculations

Widely used in the decomposition of radiative changes, radiative kernels are a set of precomputed partial derivatives representing the sensitivity of radiation to a perturbation in a specific state variable. Following (*55*) and other work cited herein, each of these sensitivities can be diagnosed as the product of a radiative kernel ($K^{x}$) and the actual change in variable *x*

$$\Delta R=\sum \Delta R_{x}\approx \sum \left(\frac{\mathrm{\partial R}}{\partial x}\times \Delta x\right)\approx \sum \left(K^{x}\times \Delta x\right)$$ (9)

where a clear-sky and all-sky *K^x^* allows one to diagnose clear-sky and all-sky $\Delta R_{x}$, respectively. The kernel-derived estimates of $\Delta R_{x}$ are also used to diagnose the aerosol direct radiative forcing as a residual calculation. Namely, the clear-sky aerosol direct forcing is estimated as the difference between the clear-sky ΔASR and the sum of the clear-sky, SW $\Delta R_{x}$ terms. Then, the all-sky IRF presented in this work is derived by scaling the clear-sky IRF by a constant that accounts for cloud masking (*11*, *55*).

Each of these terms can be diagnosed from satellite observations over the CERES period. We extend previous estimates of these terms by (*11*), similarly applying CloudSat-based radiative kernels (*56*) to satellite-observed anomalies of surface albedo from CERES-EBAF Edition 4.2 and water vapor and temperature anomalies from AIRS-CLIMCAPS V2 (*57*) to diagnose the non-cloud radiative responses. CERES-EBAF Edition 4.2 is a more recent edition than the one used in (*11*), and CLIMCAPS serves an alternative to the since-discontinued AIRS V6 operational product. As in (*11*), the clear-sky IRF is diagnosed as the difference between clear-sky net radiative anomalies from CERES and the sum of clear-sky radiative responses. Then, the all-sky IRF presented in this work is derived by scaling the clear-sky IRF by a constant that accounts for cloud masking (*11*, *55*).

#### Calculating confidence interval and trend analysis

Trends are calculated on the basis of time series of annual data, and the slope is multiplied by 10 to get a decadal trend. We use the ordinary least square fit to get the slope. The uncertainty of the trend is determined by the ratio between estimated variance of the slope and variance of the residual of the fit.

The uncertainty range for each data point for ERF_ACI_ includes the uncertainty introduced by different cloud adjustment functions. The uncertainty range for the F25 estimates is determined by the Monte Carlo sampling method in (*17*). For the $F_{\text{top}}$, the uncertainty range includes the estimated uncertainty due to CERES calculations (*16*).

We note that the uncertainty of the trend scales with both the point estimate of the uncertainty range, represented by SD σ, and $N^{-1.5}$, where *N* is the number of samples in an evenly spaced time series. In our study, *N* is 252 (monthly) and it outweighs the impact of the uncertainty range itself. The small magnitude in the uncertainty of the trend should not be taken as the uncertainty range itself being small. The uncertainty range of the trend reported in this study is statistical. This estimate of the uncertainty of slope is appropriate for accounting for the structural uncertainty in our study. If we assume that the uncertainty at each data point is random and therefore independent of each other, the uncertainty of the slope scales with the uncertainty of point estimate. For example, the uncertainty of the ERF_ACI_ trend would be 0.1 W m^−2^ decade^−1^ instead of 0.07.
