# Supplementary material for: Forcing closure suggests stronger aerosol forcing and higher climate sensitivity than the consensus
Source: Sci Adv. 2026 Oct 16;12(42):eaeh6401. doi: 10.1126/sciadv.aeh6401 (PMC13674507; doi:10.1126/sciadv.aeh6401)
Supplement: Supplementary file 1 [file sciadv.aeh6401_sm.pdf]

Science Advances
AAAS

# Supplementary Materials for

## Forcing closure suggests stronger aerosol forcing and higher climate sensitivity than the consensus

Tianle Yuan *et al.*

Corresponding author: Tianle Yuan, tianle.yuan@nasa.gov

*Sci. Adv.* **12**, eaeh6401 (2026)
DOI: 10.1126/sciadv.aeh6401

**This PDF file includes:**

Figs. S1 to S6

# Supplementary Text

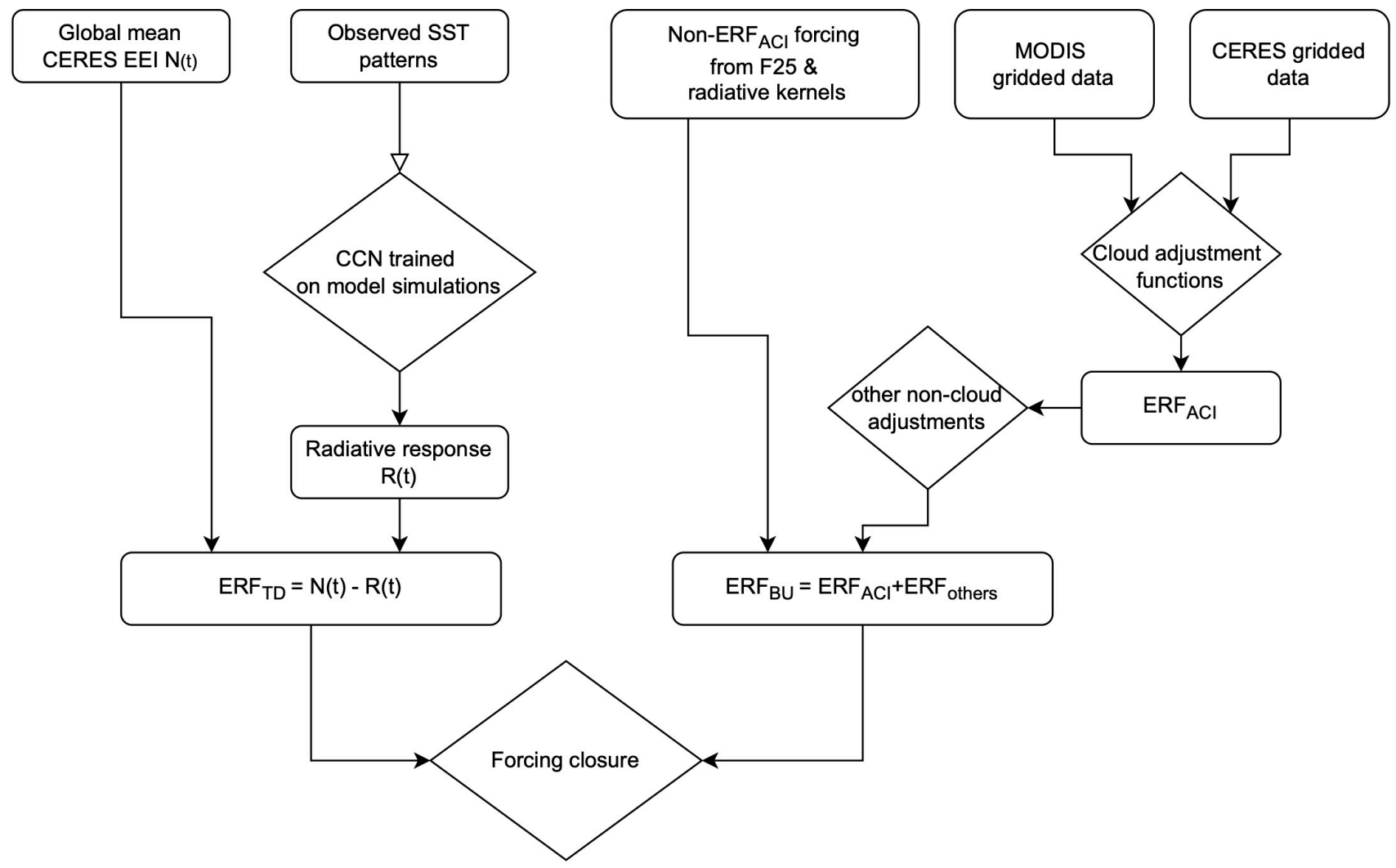


**Figure S1**: **Illustrative diagram of top-down and bottom-up methods** Illustration of the forcing closure between top-down (left path) and bottom-up (right path) methods. While there is an overlap in the dataset employed, the methods themselves are independent. The CCN is trained on internal variability of model simulations to be specific.

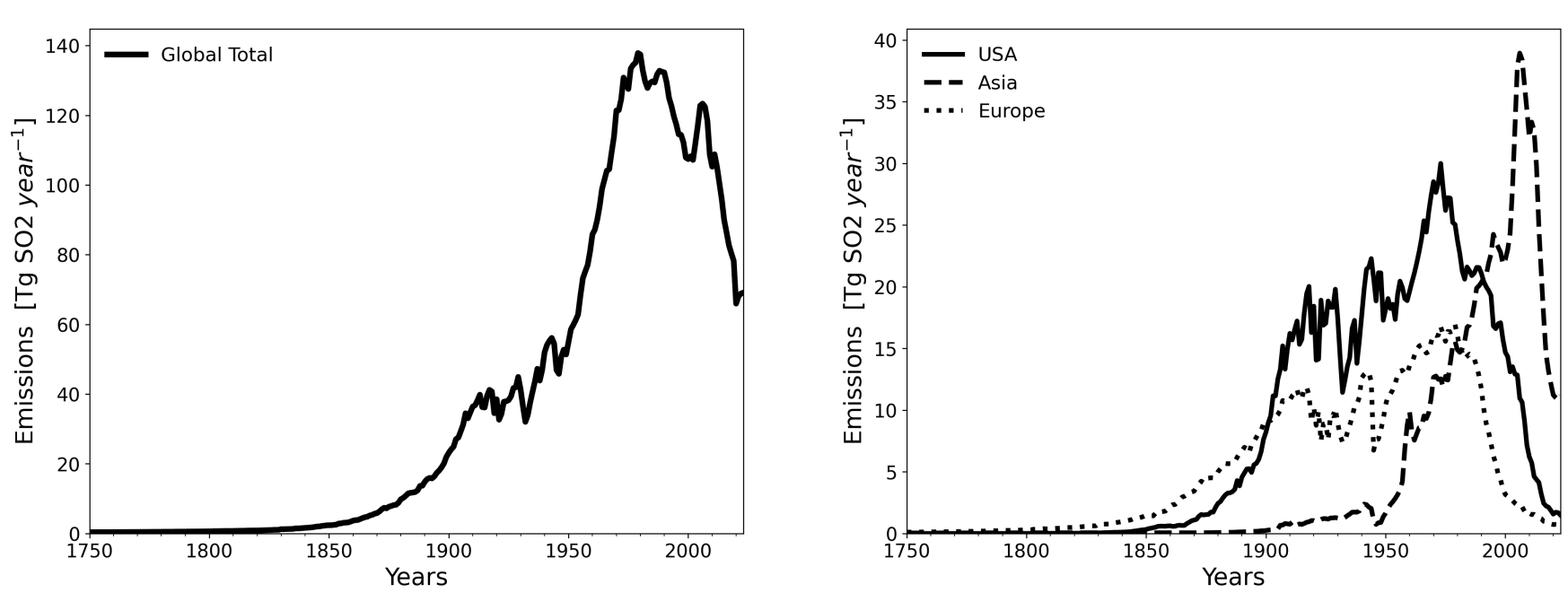


**Figure S2**: **Time series of total and regional $SO_2$ emissions** Left: time series of global $SO_2$ emissions since 1750 (*30*)). During our study period, 2003-2023, the strong decline in $SO_2$ emission and anthropogenic sulfate strongly affected $ERF_{ACI}$. Current $SO_2$ emissions are similar to those in the early 1950s.; Right: time series of $SO_2$ emissions for Europe, the United States, and East Asia (including China, South Korea, and Japan. These three regions are upwind of North Pacific and North Atlantic where strong downward $N_d$ trends are observed. The decline rate of $SO_2$ emissions for the US and East Asia is clear.

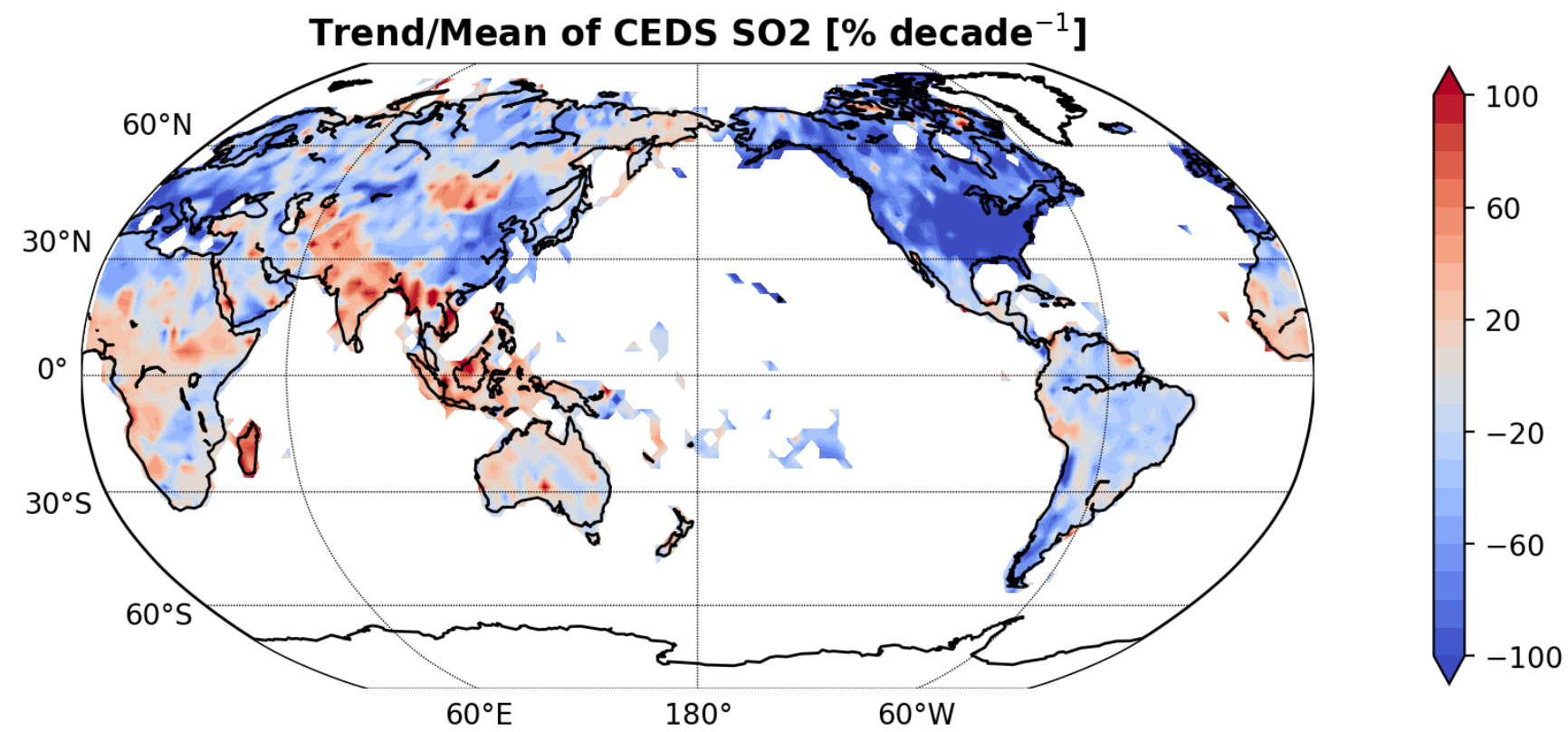


**Figure S3**: **Maps of relative emission trends based on CEDS data (*30*).** Map shows the relative trend, $\frac{Emission_{\Delta SO_2}}{Emission_{SO_2}}$ for $SO_2$ emissions. North America and Western Europe experienced drastic reductions, many times greater than 80%.

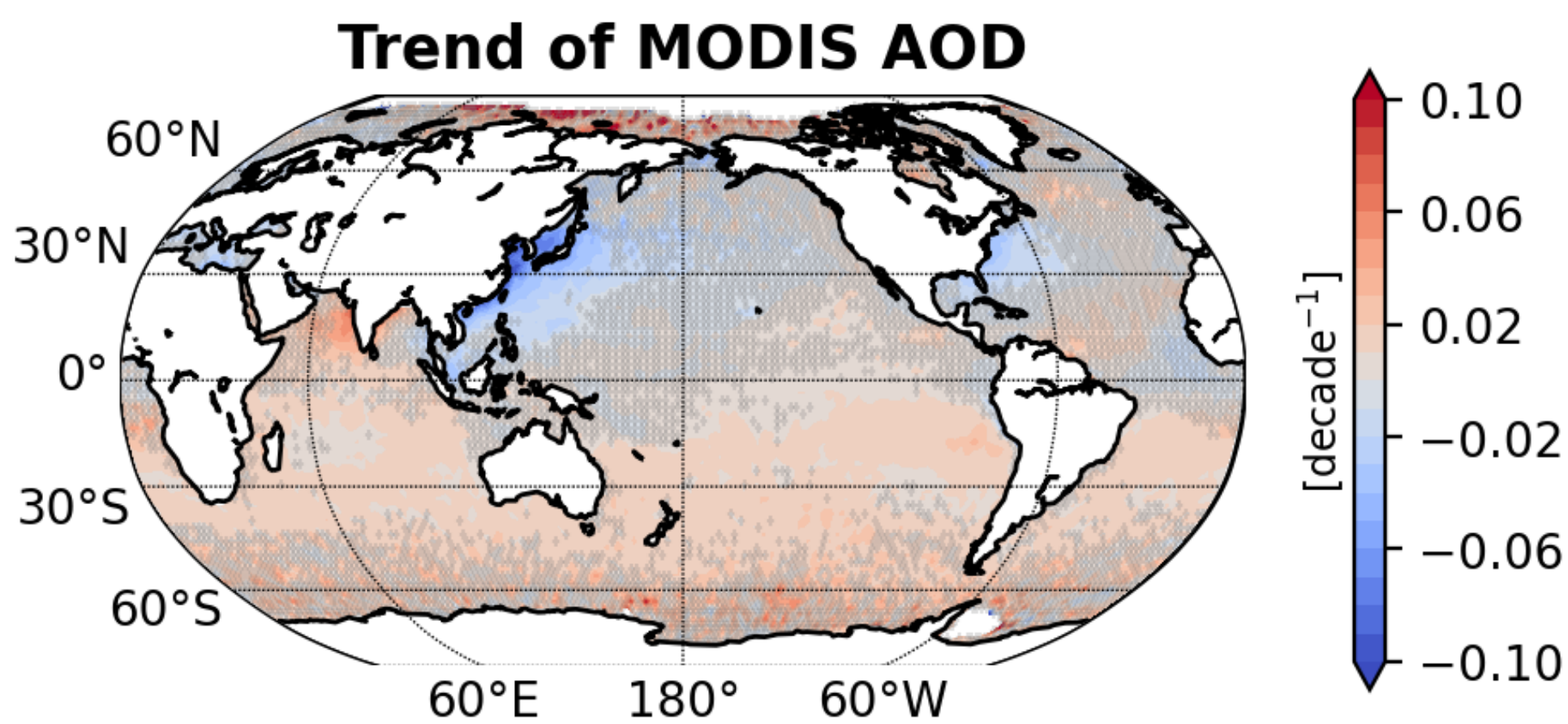


**Figure S4**: **Trend map of MODIS aerosol optical depth.** The map is broadly consistent with $N_d$ map in Figure 1b and $SO_2$ emission map in Figure 1a.

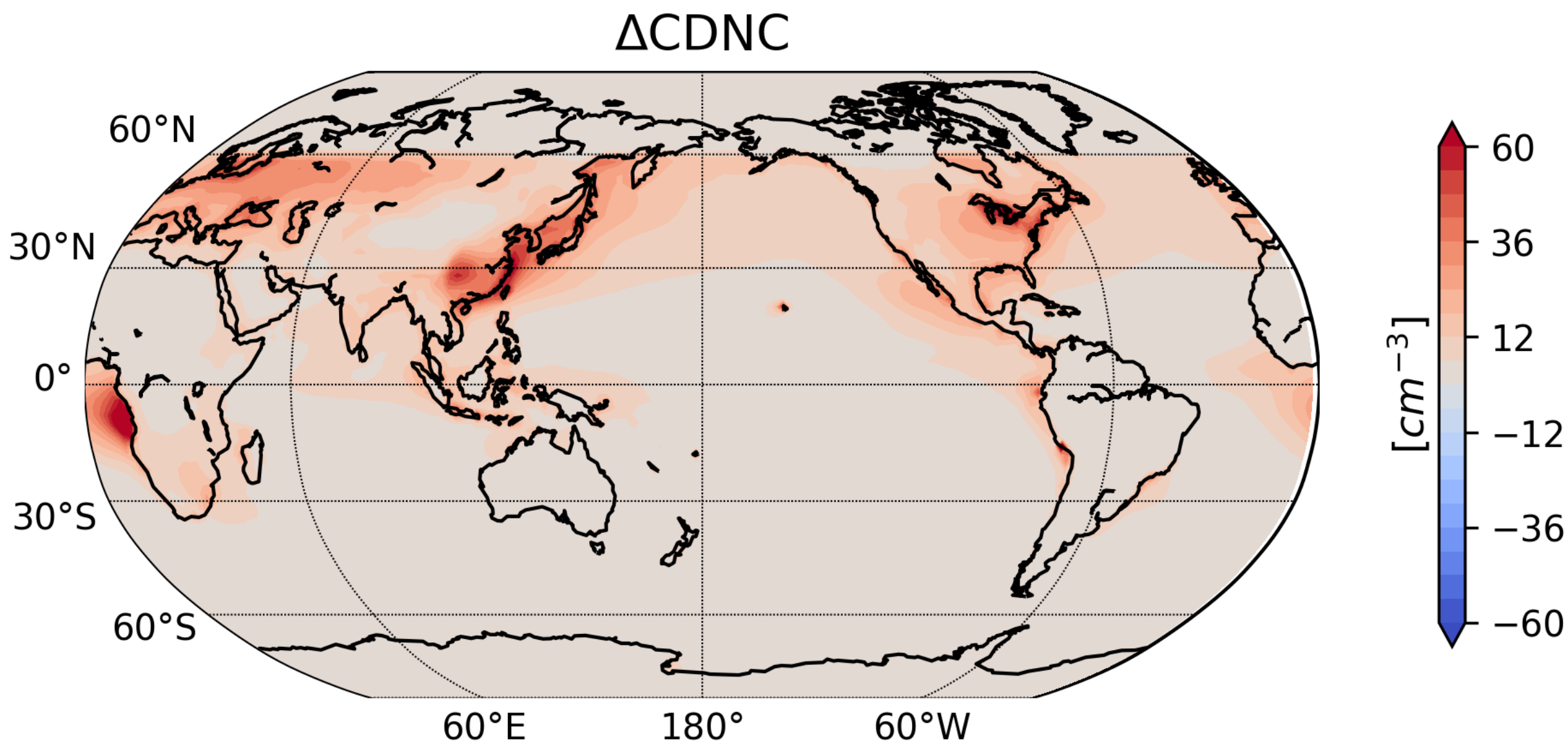


**Figure S5**: **Map of $\Delta N_d$ between 2010 and 1850.** We convert the $\Delta AOD$ from (*27*) to $\Delta N_d$ following similar procedure in (*29, 35*) and render the data into this plot. This is coupled with cloud adjustment functions to calculate $\Delta ERF_{ACI}$ between 2010 and 1850.

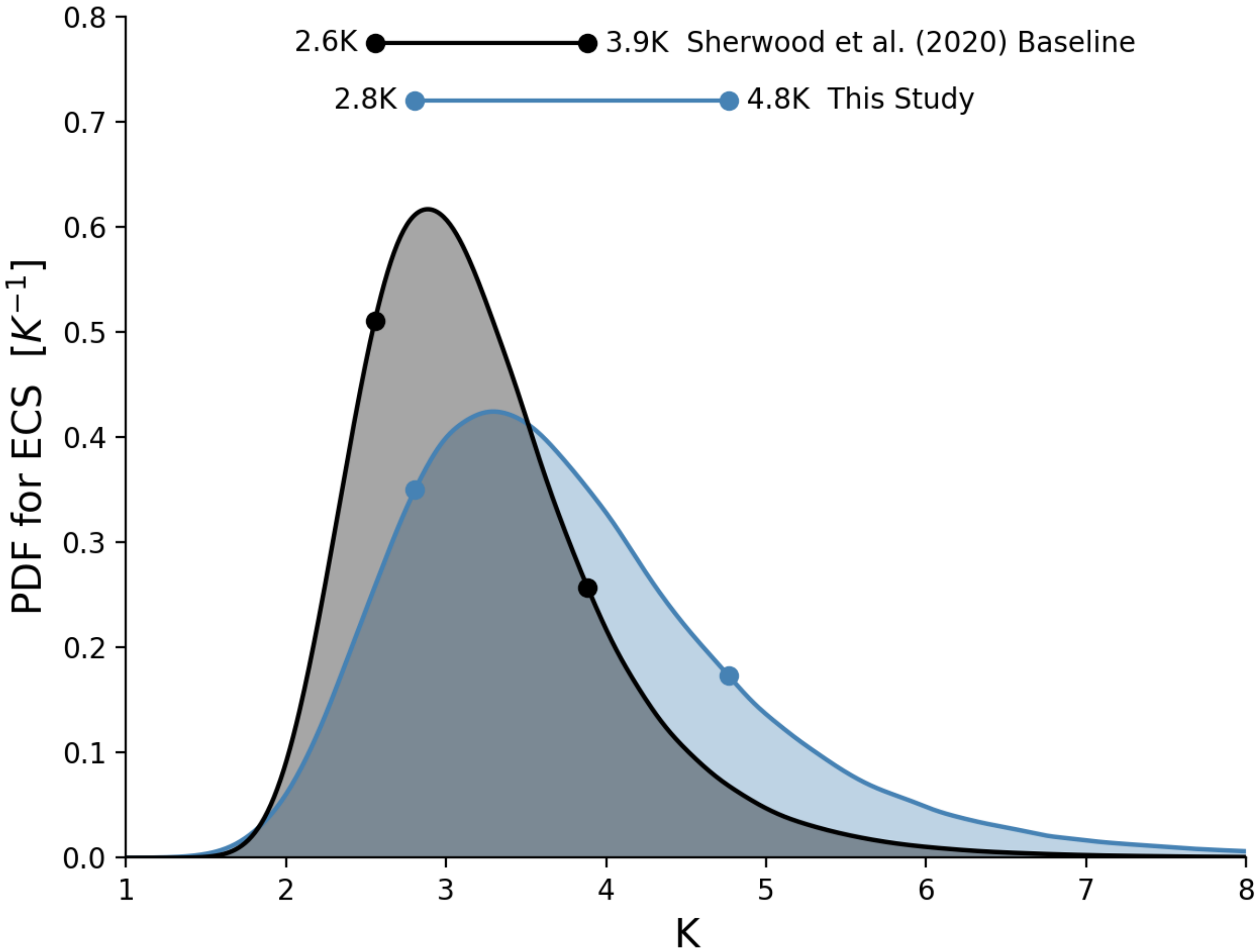


**Figure S6**: **PDFs of ECS** The figure shows the PDF of ECS from the (*14*) and our study using our updated $ERF_{ACI}$ estimates.
